# Analysis of Colorectal Cancer Gene Mutations and Application of Long Blocker Displacement Amplification Technology for High-Throughput Mutation Detection

**DOI:** 10.3390/bios15050308

**Published:** 2025-05-12

**Authors:** Ping Lu, Xinglei Su, Sirui Leong, Xuehao Xiu, Ping Song, Junjie Peng, Yunpei Si

**Affiliations:** 1 Department of Colorectal Surgery, Fudan University Shanghai Cancer Center, Fudan University, Shanghai 200032, China; hebenlu@163.com; 2 Department of Oncology, Shanghai Medical College, Fudan University, Shanghai 200032, China; 3 School of Biomedical Engineering, Zhangjiang Institute for Advanced Study and National Center for Translational Medicine, Shanghai Jiao Tong University, Shanghai 200240, China; suxinglei@shu.edu.cn (X.S.); jack23@sjtu.edu.cn (S.L.); xiuxuehao@sjtu.edu.cn (X.X.); songpingsjtu@sjtu.edu.cn (P.S.); 4 Shanghai Key Laboratory for Nucleic Acid Chemistry and Nanomedicine Institute of Molecular Medicine Renji Hospital School of Medicine, Shanghai Jiao Tong University, Shanghai 200127, China; 5 School of Life Sciences, Shanghai University, Shanghai 200444, China

**Keywords:** colorectal cancer, KRAS mutation, LBDA technology, rapid detection, bioinformatics

## Abstract

Genetic mutation detection for colorectal cancer (CRC) is crucial for precision diagnosis and treatment, yet current methods often suffer from challenges such as low sensitivity, time consumption, and high costs. In our preliminary bioinformatic analysis of 751 CRC cases from The Cancer Genome Atlas and 131 Chinese patient samples, APC, TP53, and KRAS were identified as the most frequently mutated genes. Among them, KRAS missense mutations emerged as key diagnostic biomarkers. In this study, we applied a fluorescence-based long block displacement amplification (LBDA) sensing method for the rapid, high-throughput, and cost-effective detection of KRAS genetic mutations. In the LBDA system, SYBR Green dye binds to the amplified double-stranded DNA, generating a fluorescence signal that directly reflects the abundance of mutant types (MTs). This real-time signal output enables the enrichment and sensitive detection of MTs, establishing LBDA as an efficient biosensing platform for KRAS genotyping. Using this technique, a detection limit of 0.08% variant allele frequency was achieved with 20 ng of synthetic DNA input. To evaluate clinical performance, the LBDA method was applied to 118 tissue samples from 59 CRC patients, including tumor and matched peritumoral tissues. For 59 CRC tumor samples, LBDA successfully identified KRAS mutations in 37.29% of cases, closely matching results (42.37%) obtained by next-generation sequencing and achieving 88% sensitivity and 100% specificity. In conclusion, this study presents a rapid and cost-effective mutation detection method based on optical biosensing, offering strong potential for advancing personalized CRC diagnosis and treatment.

## 1. Introduction

Colorectal cancer (CRC) is the third most common malignant tumor globally and the second leading cause of cancer-related death [1,2,3]. In China, CRC ranks second in incidence and fourth in mortality, following lung cancer, with both rates steadily increasing [4,5,6]. Advancements in medical research have highlighted the critical role of genetic mutations in CRC development and progression [7]. These genetic mutations frequently affect multiple signaling pathways, with mutations in genes such as APC, TP53, and KRAS widely acknowledged as the main drivers of CRC [8,9]. Among these, KRAS mutations are particularly critical for both the onset and prognosis of CRC. It has been reported that KRAS mutations occur in approximately 27% to 43% of CRC patients, with common mutation hotspots located in codons 12 and 13 of exon 2, including notable mutations of G13D, G12D, G12A, G12V, G12S, G12R, and G12C [10,11]. The detection of these mutations is not only essential for CRC diagnosis but also for genotyping and therapy. For example, epidermal growth factor receptor (EGFR) inhibitor cetuximab is commonly used as a first-line targeted therapy for CRC [12,13]. However, its effectiveness is limited to patients with wild-type (WT) KRAS genes, and it does not benefit those with mutant KRAS genes. Therefore, KRAS genotyping, which detects KRAS gene mutation status, is crucial for the individualized treatment of CRC, as it helps guide targeted therapy and predict treatment response.

Numerous studies have investigated the relationship between mutations in the above key driver genes and CRC metastasis [14,15,16,17]. In our study, we compared genetic mutations in a cohort of 131 Chinese CRC patients, including 120 CRC tissue samples and 11 blood samples with data from 751 CRC tissue samples in the Cancer Genome Atlas (TCGA) dataset to examine the differences in driver gene mutations across populations and their potential implications. Building on this broad analysis of CRC mutations, we further investigate detection methods for specific driver mutations, such as those in the RAS gene, which play a crucial role in CRC progression.

Current methods for RAS gene mutation detection include next-generation sequencing (NGS) and quantitative real-time polymerase chain reaction (qPCR). NGS is widely utilized in clinical settings for its comprehensive analysis but typically requires a longer turnaround time (approximately one week) and incurs higher costs [18,19,20,21]. In contrast, traditional qPCR offers faster results but has limitations in sensitivity and throughput, particularly when identifying low-frequency mutations.

To address these challenges, we employed the long blocker displacement amplification (LBDA) strategy, a qPCR-based method with high sensitivity for detecting any single nucleotide variants, even dual mutations, within the nonhomologous region targeted by the blocker [22,23]. This technique utilizes a WT-specific nucleic acid blocker that binds WT templates with higher affinity, thereby suppressing their amplification; in contrast, mismatches between the blocker and mutant-type (MT) templates reduce hybridization stability, allowing the forward primer (FP) to displace the blocker and amplify MT sequences. In the LBDA system, SYBR Green dye intercalates into the accumulating double-stranded DNA products, producing a fluorescence signal that correlates with the abundance of MT templates. This real-time, mutation-enriched detection strategy makes BDA a powerful and efficient mutation biosensing platform.

In this study, we first conducted a bioinformatic analysis of gene alterations in CRC using data from TCGA (*n* = 751) and a Chinese patient group (*n* = 131). Mutation genes such as TP53, APC, KRAS, and NRAS were commonly observed in both patient populations, providing significant diagnostic and prognostic values. The most prevalent KRAS mutations identified were G12D, G12V, and G13D, which correlated with a notably lower five-year survival rate for RAS-mutant patients, particularly in advanced stages. To validate the clinical applicability of BDA, we further applied our LBDA-based biosensing method to 59 CRC patients, including 59 tumor samples and the corresponding peritumoral tissue samples. LBDA successfully identified KRAS mutations in 37.29% of tumor samples, achieving 88% sensitivity and 100% specificity compared to the NGS method, demonstrating its high accuracy, sensitivity, and translational potential for clinical CRC mutation detection.

## 2. Materials and Methods

### 2.1. Bioinformatics Analysis of CRC Patients

In this study, we performed bioinformatics analysis on the sequencing data of CRC patients from both the TCGA cohort and the Chinese group. The TCGA group consisted of 751 CRC patients, with genomic data obtained through whole-exome sequencing (WXS) of tissue samples downloaded from the TCGA database (Available online: https://www.cancer.gov/ccg/research/genome-sequencing/tcga (accessed on 1 July 2024)). The Chinese group included 131 CRC patients (120 tissue samples and 11 blood samples), with WXS data obtained from Fudan University Shanghai Cancer Center.

Bioinformatics analysis was performed using R (version 4.4.1). The maftools software (version 2.10.0) was used to display the gene variation distribution of each model, and the comparisons between each model were shown using VennDiagram (version 1.7.3). ggplot2 (version 3.5.1) was used to analyze the mutational frequencies and survival rate. Further analysis of mutational frequencies and KRAS-associated survival rates was performed to provide deeper insights into CRC genetic mutations.

### 2.2. Design of KRAS Templates, Primers, and Probes

The target gene of KRAS WT and MT templates, harboring a G > T substitution at the rs121913535 locus, were separately constructed on two PUC-SP plasmids. The synthesized KRAS target gene sequences are shown in Table 1. All primers and probes used for KRAS mutation detection were designed according to the principles outlined in the LBDA method [23]. The sequences of the FP, reverse primer (RP), and blocking probe are presented in Table 2.

### 2.3. qPCR Experimental Protocol

All DNA oligonucleotide sequences used in this study, including primers, blocker probes, as well as WT and MT plasmids, were custom synthesized by Sangon Biotech (Shanghai) Co., Ltd. (Shanghai, China), and stored in 1 × TE buffer with low EDTA.

For DNA samples extracted, qPCR detection was performed using a 96-well plate on the CFX Opus96 Real-Time PCR Detection System, with reaction conditions based on LBDA protocols. Each 10 μL qPCR reaction without blocker (NB) consisted of 4.6 μL double-distilled water (ddH_2_O), 2 μL Q5 Reaction Buffer, 1 μL each of upstream and downstream primers (4 μmol/L), 0.2 μL of 10 mM dNTP, 0.1 μL Q5 high-fidelity DNA polymerase, 0.1 μL SYBR Green, and 1 μL template, while the reaction with blocker (WB) contained 3.6 μL ddH_2_O, 2 μL Q5 Reaction Buffer, 1 μL each of upstream and downstream primers (4 μmol/L), 1 μL of 20 μM blocker, 0.2 μL of 10 mM dNTP, 0.1 μL Q5 high-fidelity DNA polymerase, 0.1 μL SYBR Green, and 1 μL template. The final concentration of primers and blockers used in each reaction was between 400 nM and 2 μM. The qPCR running procedure was as follows: 98 °C for 30 s, followed by 50 cycles of (98 °C for 10 s, 70 °C for 30 s, 72 °C for 3 min).

### 2.4. Standard Curve Establishment

The KRAS MT and WT templates were first diluted to 6000 copies/μL in 1 × TE buffer. To establish a standard curve, KRAS MT and WT templates were mixed in varying ratios to generate templates with different variant allele frequencies (VAFs) ranging from 0.08% to 100%. For each VAF, two qPCR reactions were performed, including a 10 μL reaction NB system and a system WB. The NB reaction system consisted of 4.6 μL ddH_2_O, 2 μL Q5 Reaction Buffer, 1 μL each of FP and RP (4 μmol/L), 0.2 μL of 10 mM dNTP, 0.1 μL Q5 high-fidelity DNA polymerase (2 U/uL), 0.1 μL SYBR Green, and 1 μL of the plasmid template. The WB reaction system contained a mixture of 3.6 μL ddH_2_O, 2 μL Q5 Reaction Buffer, 1 μL each of FP and RP (4 μmol/L), 1 μL of 20 μM blocker, 0.2 μL of 10 mM dNTP, 0.1 μL Q5 high-fidelity DNA polymerase, 0.1 μL SYBR Green, and 1 μL template. For each VAF, the Ct values from two reaction systems were used to calculate the ΔCt value (Ct_NB_−Ct_WB_). Then, a standard curve was generated by plotting the ΔCt values against the logarithmic VAFs, followed by linear fitting using Origin 2025 software.

### 2.5. Tissue DNA Extraction and KRAS Mutation Detection Using LBDA Technology

A total of 118 tissue samples, including tumor tissues and their matched peritumoral tissues from 59 CRC patients, were collected from the Department of Colorectal Surgery, Fudan University Shanghai Cancer Center, between 2023 and 2024. These samples were used to establish and validate the KRAS mutation detection method. For all frozen tissues (tumor and peritumoral), DNA extraction was performed using the MolPure^®^ Cell/Tissue DNA Kit, strictly following the kit’s manual to ensure DNA integrity and purity. In the final extraction step, a two-step elution method was employed using 60 μL and 50 μL of elution buffer, respectively, to maximize DNA recovery from the columns. All extracted DNA samples were stored at −20 °C. To validate the detection applicability of our LBDA approach, we further applied the qPCR-based LBDA technology to CRC patients’ tissue samples according to the qPCR experimental protocol.

## 3. Results

### 3.1. Genetic Alterations in TCGA

**Figure 1:**
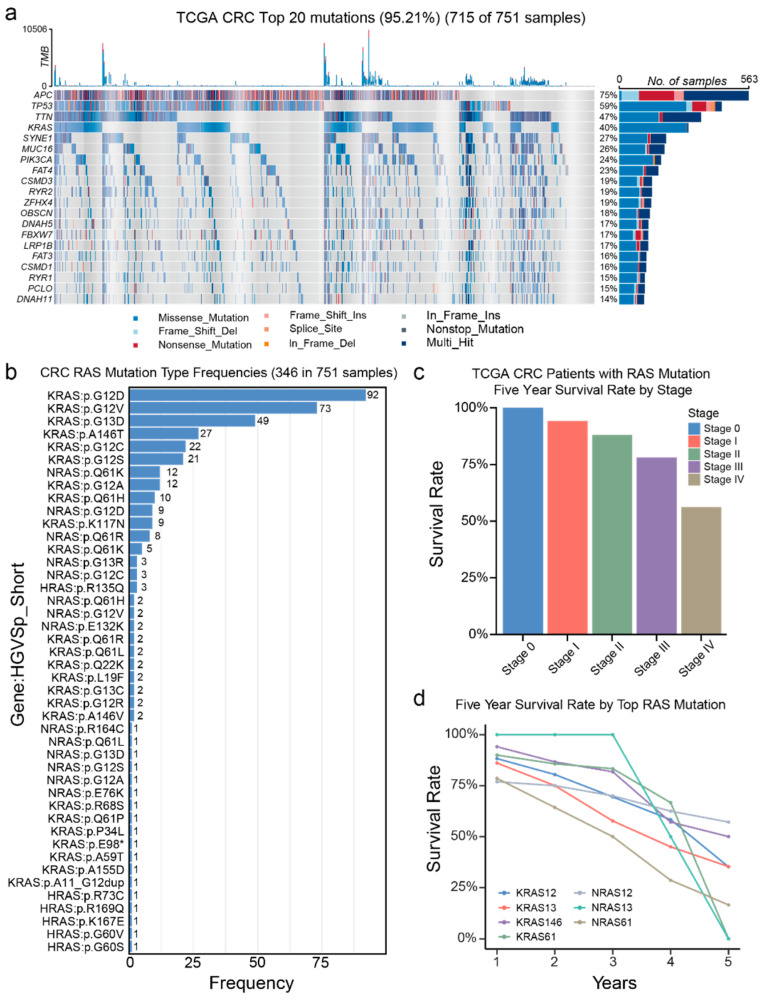
Analysis of gene mutations in CRC tissue samples. The data are obtained from TCGA. (**a**) Mutation frequencies of the top 20 genes in 751 CRC patients. (**b**) Distribution of RAS gene mutation types among CRC patients in the TCGA database. (**c**) Survival rates of CRC patients with RAS mutation at different stages from the TCGA database. (**d**) Five-year survival rates of CRC patients with high-frequency RAS mutations, specifically in KRAS (codons 12, 13, and 146) and NRAS (codons 12, 13, and 61).

To gain further insight into gene mutational patterns in CRC tissue samples, we analyzed the clinical sample information of patients in TCGA and summarized the results in Figure 1. We identified the top 20 most frequently mutated genes, with the top 4 being APC, TP53, TTN, and KRAS, found in 75%, 59%, 47%, and 40% of TCGA cases, respectively (Figure 1a). Among these genes, APC is a tumor suppressor that regulates the Wnt signaling pathway. Functional APC degrades β-catenin, preventing excess cell growth. Mutations in APC disrupt this process, causing β-catenin accumulation and leading to uncontrolled proliferation, a hallmark of CRC and familial adenomatous polyposis (FAP) [24]. TP53 encodes p53, the “guardian of the genome”, which controls cell cycle arrest and apoptosis. Mutations in TP53, found in over 50% of cancers, impair these functions, allowing genetic instability [25,26]. TTN encodes titin, a protein essential for muscle function. Although primarily linked to cardiomyopathies, TTN mutations frequently appear in lung adenocarcinoma, CRC, and melanoma. They are often considered passenger mutations due to the gene’s large size but may still influence tumor behavior [27,28]. KRAS encodes a GTPase protein controlling the RAS/MAPK pathway for cell proliferation [29]. Mutations at codons 12, 13, and 61 keep KRAS permanently active [30].

Given the prevalence of RAS mutations at codons 12, 13, and 61, we further analyzed the frequencies of RAS mutation types in 751 TCGA cases (Figure 1b). RAS mutations were found in 46.07% (346/751) of CRC cases, consistent with reported frequencies of approximately 40% [10]. The most frequent RAS mutation types were KRAS: G12D (26.59%, 92/346), KRAS: G12V (21.10%, 73/346), KRAS: G13D (14.16%, 49/346), KRAS: A146T (7.80%, 27/346), and KRAS: G12C (6.36%, 22/346). We also assessed the 5-year survival rates of TCGA cases with RAS mutations by stage (Figure 1c) and by the top RAS mutation types (Figure 1d). The overall 5-year survival rate by stage was 94.23% in stage I, 88% in stage II, 78.08% in stage III, and 56.25% in stage IV. Notably, the 5-year survival rates for cases with the most common RAS mutations significantly decreased, falling below 50% for five types of RAS mutations.

### 3.2. Genetic Alterations in a Chinese CRC Patient Group

**Figure 2:**
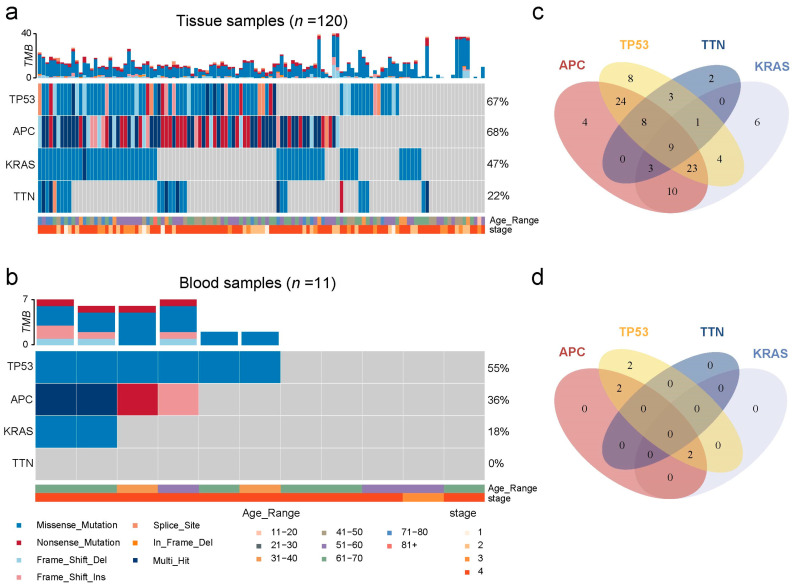
Mutation analysis of CRC tissue (*n* = 120) and blood (*n* = 11) samples based on the NGS results from a Chinese patient population. Distribution of TP53, APC, KRAS, and NRAS mutations in CRC tissue (**a**) and blood (**b**) samples. Mutational profiles of TP53, APC, KRAS, TTN, and NRAS in CRC tissue (**c**) and blood (**d**) samples.

To further investigate the gene mutations in CRC patients within a Chinese patient population, we analyzed genetic variations in 131 patients, including 120 tissue samples and 11 blood samples (Figure 2 and Figure S1). The top four mutated genes include TP53, APC, KRAS, and TTN, and they were detected in 87.50% (105/120) of CRC tissue samples (Figure 2a) and 54.55% (6/11) of CRC blood samples (Figure 2b). In tissue samples, 127 APC mutations were identified in 81 cases, with nonsense mutations being the most common (71, 55.91%). For TP53, 86 mutations were detected in 80 cases, predominantly missense mutations (57, 66.28%). KRAS showed 58 mutations in 56 cases, with missense mutations (57, 98.28%) as the most dominant. TTN mutations were observed in 26 cases, totaling 33 mutations, of which 30 (90.91%) were missense mutations (Figure 2a). In blood samples, six missense mutations of TP53 were observed in six cases; six APC mutations were found in four cases, with nonsense mutations being the most common (3, 50%); KRAS mutations were observed in two cases, both being missense mutations, while no TTN mutations were detected (Figure 2b). These results highlight the significance of KRAS mutations, as they exhibit both high prevalence and a strong preference for missense mutations, which often activate oncogenes and drive cancer progression [31,32]. In contrast, genes with nonsense mutations or other mutations may result in loss of function rather than direct oncogenic activation and, therefore, may not actively contribute to cancer development [33].

The mutation patterns of the top mutated genes in CRC patient tissue and blood were summarized in Figure 2c,d. In tissue samples, 20% (24/120) of cases exhibited concurrent mutations in both TP53 and APC, 3.33% (4/120) showed concurrent mutations in TP53 and KRAS, 8.33% (10/120) had mutations in both APC and KRAS, 19.17% (23/120) displayed mutations in TP53, APC, and KRAS together, and 7.5% (9/120) had mutations in TP53, APC, KRAS, and TTN (Figure 2c). In blood samples, the most common mutation combination was APC and TP53 (18.18%, 2/11), followed by the combination of APC, TP53, and KRAS (Figure 2d). The observed discrepancies in genetic alterations between tissue and blood samples may primarily result from the limited number of blood samples analyzed, leading to potential statistical bias. In addition, biological differences, such as the heterogeneous release of circulating tumor DNA (ctDNA) from primary and metastatic lesions, and the inherently lower abundance of ctDNA compared to tissue-derived DNA, could further contribute to these variations. Therefore, expanding the blood sample cohort and conducting comprehensive analyses are necessary to substantiate these preliminary findings.

In conclusion, our analysis of gene mutations in CRC tissue and blood samples from a Chinese population identified TP53, APC, and KRAS as the top three frequently mutated genes, covering a significant proportion of CRC cases, highlighting their potential as diagnostic markers. Additionally, mutation combinations involving TTN further expand our understanding of the CRC mutational landscape. Notably, 98.28% of KRAS mutations were missense, which play a crucial role in cancer development and progression, making them valuable potential biomarkers.

### 3.3. Comparison of the Mutation Gene Signatures Between TCGA and Chinese Patient Population

**Figure 3:**
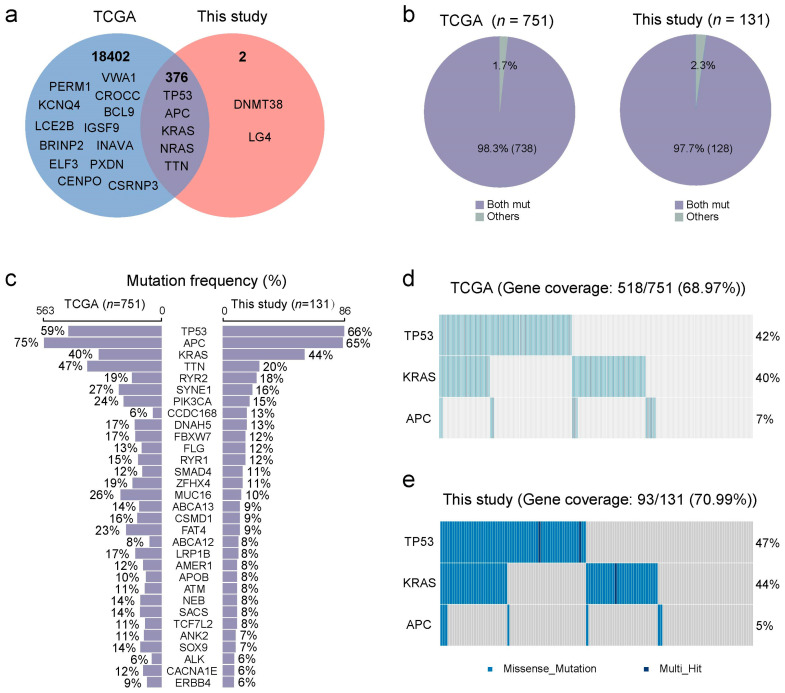
Consistency analysis of gene mutations in CRC patients based on the TCGA database and NGS results from a Chinese patient population in this study. (**a**) Mutant genes in TCGA data with those identified in the NGS results from a Chinese patient population. (**b**) Coverage of patients with shared mutated genes in both the TCGA dataset and this study. (**c**) Comparison of high-frequency mutant gene frequencies between the TCGA dataset and this study. Missense mutation distribution of TP53, APC, and KRAS genes from the TCGA dataset (**d**) and this study (**e**).

To explore the similarities and differences in gene mutation patterns between the TCGA and the Chinese patient population, we compared the gene mutations in CRC patients from both groups (Figure 3 and Figure S2). A total of 376 similar mutations, including TP53, APC, KRAS, NRAS, and TTN, were identified in both patient populations. These 376 mutations occurred in 98.4% (738/751) of CRC patients in the TCGA patient population and 97.7% (128/131) of CRC patients in the Chinese patient population (Figure 3b). However, 18,402 mutations found in TCGA were not present in the Chinese patient population, while 2 mutations unique to the Chinese patient population were found (Figure 3a).

We further compared the mutational frequencies between the TCGA and the Chinese patient population (Figure 3c–e). The top four mutated genes in both patient populations were TP53, APC, KRAS, and TTN in both databases. In the TCGA patient population, mutation rates for TP53, APC, KRAS, and TTN were 59%, 75%, 40%, and 47%, respectively, compared to 66%, 65%, 44%, and 20% in the Chinese patient population (Figure 3c). This reveals a similar KRAS mutation rate in both patient populations, along with a notable difference in the mutation rates of three other genes, consistent with the findings of other Chinese studies [34,35]. In addition, we analyzed the frequency of missense mutations in TP53, APC, and KRAS genes across both patient populations. In the TCGA patient population, missense mutations were observed in 317 cases of TP53 (42.21%), followed by KRAS with 299 cases (39.81%), and APC with 54 cases (7.19%) (Figure 3d). Similarly, in the Chinese patient population, missense mutations were detected in TP53 (61 cases, 46.56%), KRAS (58 cases, 44.27%), and APC (7 cases, 5.34%). The comparative analysis results indicate that KRAS mutation frequencies and patterns were similar in both patient populations, suggesting that KRAS could serve as genetic biomarkers across ancestries.

### 3.4. Detection of KRAS Mutations Using LBDA Technology

**Figure 4:**
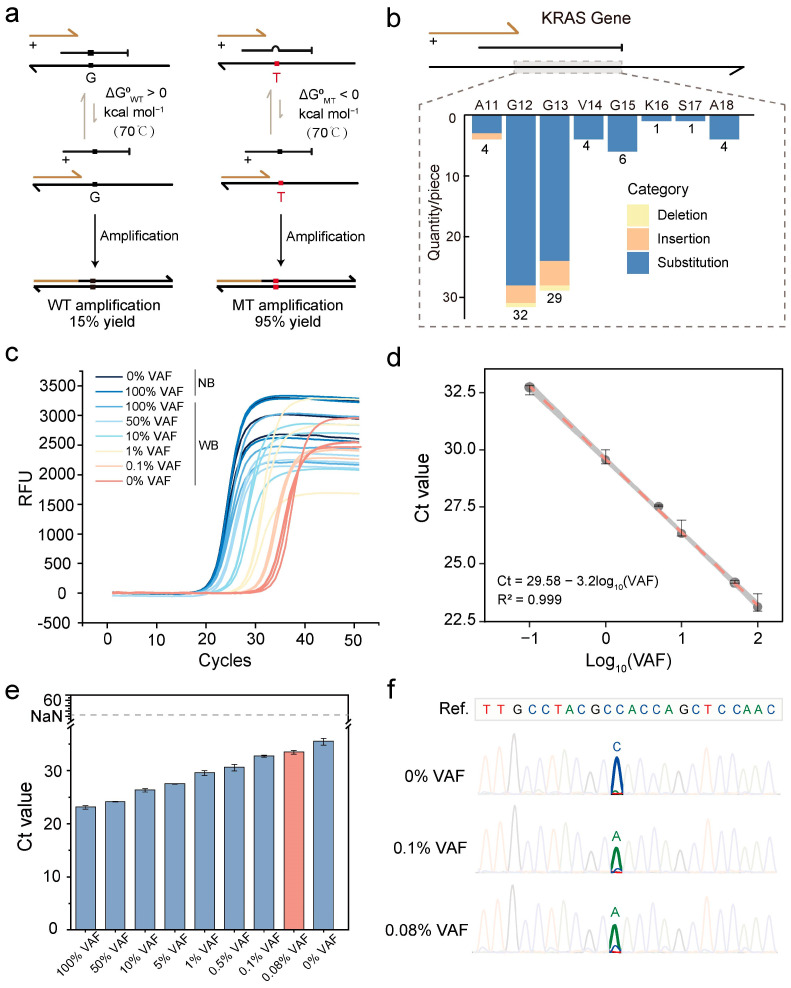
LBDA design and performance validation for high-frequency KRAS mutation sites in CRC. (**a**) Schematic diagram of WT and MT enrichment for rs121913535 (T > G) designed using LBDA. (**b**) Overview of all mutation types covered by the LBDA design blocker for the KRAS gene. Based on mutation data from the COSMIC (Catalogue of Somatic Mutations in Cancer) database, a single design covers 81 mutation types at amino acid positions 11, 12, 13, 14, 15, 16, 17, and 18 of the KRAS gene. (**c**) qPCR amplification curves of the KRAS gene with different VAFs; both experiments, WB and NB, were demonstrated. (**d**) Standard curve for KRAS mutations. A series of VAFs from 0.1% to 100% VAF were performed. (**e**) Ct values of KRAS with different VAFs obtained using the LBDA detection system in the presence of blocker. A VAF of 0.08% can still be clearly distinguished from 0% VAF with 20 ng DNA input. (**f**) Sanger sequencing results of 0%, 0.1%, and 0.08% VAF amplified by the LBDA system.

As aforementioned, KRAS is one of the most frequently mutated genes in CRC, and missense mutations are the most predominant type. These missense mutations are more likely to activate oncogenes, in contrast to the loss-of-function mutations observed in APC, thereby underscoring the importance of KRAS mutation detection as a key diagnostic marker for CRC patients. However, current KRAS mutation detection methods face challenges such as low sensitivity, limited throughput, and long detection time [23,36,37,38,39,40,41,42]. To address these issues, we employed LBDA technology to achieve high sensitivity, high throughput, and rapid detection of KRAS mutations [23,43,44]. Herein, we used a long blocker specifically targeting the KRAS gene (Figure 4a). This blocker covers multiple mutation sites within the enrichment regions, with a particular focus on codons 12 and 13 of the KRAS gene. First, we employed the synthetic DNA template rs121913535 (G) as the WT template and the variant (T) as the MT. Based on the WT sequence, we designed a long blocker that covers 81 hotspot mutation sites with a single probe, enabling high-throughput detection of KRAS mutations (Figure 4b).

Next, we evaluated the inhibitory effect of the long blocker on the aforementioned KRAS mutations and its ability to enrich different mutation types. Assay performance was validated by mixing varying ratios of WT and MT plasmids to simulate templates with different VAFs. In the NB PCR system, the Ct values of MT and WT templates were similar, whereas, in the WB PCR system, they were clearly distinguishable, indicating effective WT suppression and MT enrichment by the long blocker. Moreover, in the WB system, the Ct values for MT templates decreased progressively with increasing VAFs (Figure 4c). A standard curve was then established by plotting the Ct values of MT templates against the logarithmic VAF (LogVAF) (Figure 4d). Furthermore, the enrichment fold was calculated based on 2^^ΔCt^, where ΔCt represents the difference in Ct values between 100% and 0% VAFs. Specifically, for the designed KRAS locus, the ΔCt value was 12.35, corresponding to a 5220.6-fold enrichment. Notably, the detection limit of the system was determined to be as low as 0.08% VAF with 20 ng of synthetic DNA input (Figure 4e), which was further confirmed by Sanger sequencing results (Figure 4f).

Compared to existing KRAS mutation detection methods, the LBDA technology demonstrates significant advancements in both performance and clinical applicability [23,36,37,38,39,40,41,42]. As shown in Table 3, LBDA achieves an ultra-low detection limit of detection (LoD) of 0.08% VAF, surpassing AS-PCR, digital PCR. Compared to NGS assays, the LBDA method not only reduces the detection time by a factor of seven but also lowers costs by two orders of magnitude, making it a more cost-effective and clinically viable solution for KRAS genotyping (Figure S13). Additionally, LBDA technology surpasses traditional qPCR methods in both detection throughput and sensitivity. With a single probe capable of covering 81 hotspot mutations, it can detect low-frequency mutations as rare as 0.08% VAF, further enhancing its potential for clinical diagnostics. In summary, the LBDA-based KRAS gene detection system demonstrates excellent sensitivity, specificity, and rapid detection capabilities, positioning it as a promising tool for clinical applications.

### 3.5. Clinical Application of the LBDA Method for Detecting KRAS Mutations in CRC Tissues

**Figure 5:**
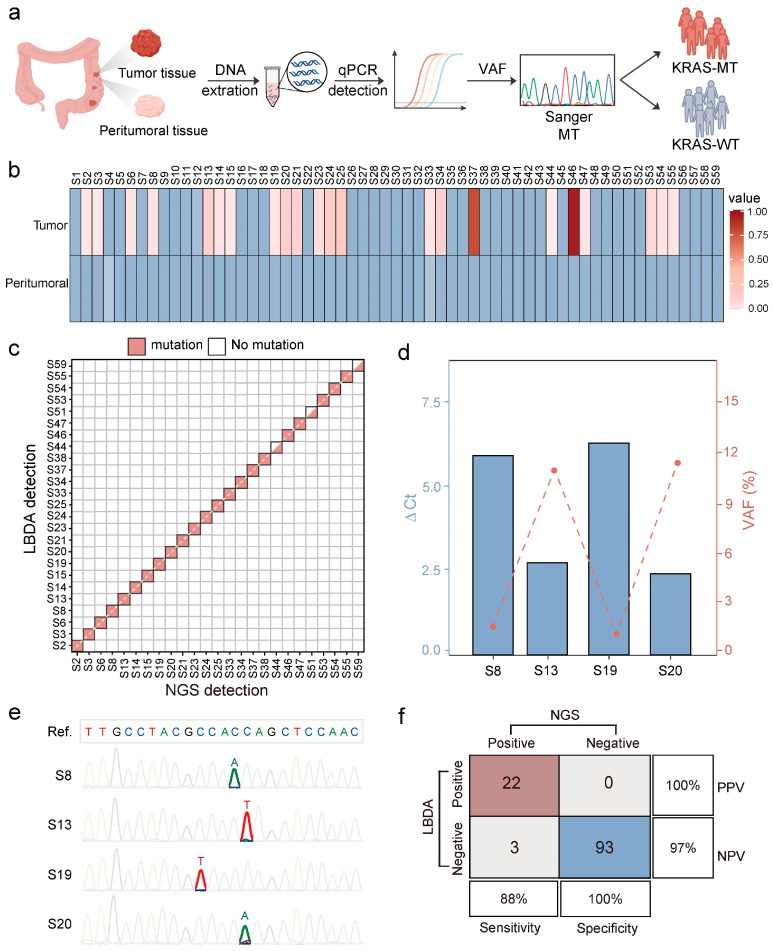
LBDA-qPCR detection of KRAS mutations in CRC tissue samples. (**a**) Overview of KRAS mutation detection in clinical tumor and peritumoral tissue samples. (**b**) Summary of mutation frequencies detected by LBDA in 118 tissue samples, including 59 tumor tissue samples and their paired adjacent tissue samples. (**c**) Comparison of LBDA and NGS detection results for KRAS gene mutations. LBDA detected 22 positive samples, while NGS detected 25 mutated samples. (**d**) The ΔCt values and VAF of the four samples. The four samples were S8, S13, S19, and S20, with ΔCt values of 5.91, 2.73, 6.27, and 2.4, respectively, with corresponding VAFs of 1.76%, 11%, 1.21%, and 11.47%. (**e**) Sanger sequencing peaks amplified by LBDA in the four samples. The mutation types were KRAS: G12V (S8), KRAS: G12D (S13), KRAS: G13D (S19), and KRAS: G12C (S20). (**f**) Confusion matrix analysis of LBDA and NGS methods. The matrix illustrates the performance indicators of the LBDA diagnostic method, including true positives (TP), false positives (FP), true negatives (TN), and false negatives (FN).

As a final validation, we applied the LBDA technology to detect KRAS gene mutations in tissue samples from 59 CRC patients, including 59 tumor samples and their corresponding peritumoral tissue samples. Initially, the extracted DNA samples were quantified using the qPCR-based LBDA method, and Sanger sequencing was carried out for those samples with detectable VAFs to confirm the mutations (Figure 5a). Among the 59 tumor tissue samples, gene mutations were detected by LBDA in 22 samples, accounting for 37.29% of the total samples. Among these, 22.73% of samples exhibited the KRAS: G12D mutation, 13.64% of samples had the KRAS: G12V mutation, and 27.27% of samples showed the KRAS: G13D mutation [10]. No mutations were found in the 59 peritumoral samples (Figure 5b and Figure S3, Table S1). We further compared the results of the LBDA test with the NGS results from the hospital. The positive agreement rate between the LBDA method and the NGS test was 88%, while the true negative rate was 100% (Figure 5c and Figures S3–S12). Furthermore, we randomly selected four samples with a VAF greater than the detection limit for Sanger sequencing to verify the mutation type (Figure 5d and Figure S4), and the sequencing results were consistent with the NGS detection results (Figure 5e). Additionally, we evaluated the diagnostic sensitivity and specificity of the LBDA method. Based on confusion matrix analysis of the 118 clinical samples, the LBDA method exhibited a sensitivity of 88%, specificity of 100%, positive predictive value (PPV) of 100%, and negative predictive value (NPV) of 97% (Figure 5f), indicating the satisfactory performance of LBDA for KRAS mutation detection in clinical samples.

## 4. Conclusions

In conclusion, we comprehensively analyzed genetic alterations in CRC using data from both the TCGA database and a Chinese patient cohort, revealing the prominent roles of KRAS, TP53, and APC mutations in CRC pathogenesis. Among these, recurrent KRAS mutations, particularly G12D, G12V, and G13D, were closely associated with poor prognosis and reduced survival, highlighting KRAS as a key diagnostic and prognostic biomarker. To address the sensitivity and detection throughput of current detection methods, we applied an LBDA sensing approach for KRAS mutation detection. This method achieved a detection limit as low as 0.08% variant allele frequency with only 20 ng of DNA input. In clinical testing using CRC tissue samples, LBDA demonstrated high sensitivity (88%) and specificity (100%), comparable to NGS. By enabling rapid, cost-effective, and high-throughput detection of KRAS mutations, LBDA represents a promising alternative to conventional approaches and offers valuable potential for advancing personalized CRC diagnostics and treatment.

## Figures and Tables

**Table 1:** Sequences of synthetic KRAS templates.

Sequence Name	Sequence
KRAS-WT	TGACTGAATATAAACTTGTGGTAGTGGAGCTGGT**G**GCGTAAGCAAGAGTGCCTTGACGATACAGCTAATTCAGAATCATTTTGTGGACGAATATGATCCAACAATAGAGGTAAATCTTGTTTTATATGCATATTACTGGTGCAGGACCATTCTTTGATACAGA
KRAS-MT G13C (G > T)	TGACTGAATATAAACTTGTGGTAGTGGAGCTGGT**T**GCGTAAGCAAGAGTGCCTTGACGATACAGCTAATTCAGAATCATTTTGTGGACGAATATGATCCAACAATAGAGGTAAATCTTGTTTTATATGCATATTACTGGTGCAGGACCATTCTTTGATACAGA

**Table 2:** Sequences of primers and blockers used for KRAS mutation detection.

Sequence Name	Sequence
KRAS-FP	5′-GCTCTTCCTCTCACATCTTTATTTAACC-3′
KRAS-RP	5′-TCCACACTGCAGTGTGAACAG-3′
KRAS-Blocker	GTAGTTGGAGCTGGTGGCGTAGGCAAGAGT/iSpC3//iSpC3/CA

**Table 3:** Comparison of LBDA with other KRAS mutation detection methods.

Method	LoD (VAF %)	Throughput (Per Test)	Mutation Coverage	Time (h)	Cost ($)	Reference
LBDA (this work)	0.08	>81	Known + novel	3	~2	[23]
AS-PCR	0.1~1	1	Known only	~5	~5	[39]
Asy-PCR/SERS	0.1	1	Known only	~5	~6	[40]
Chamber-Based Digital PCR	0.2	3	Known only	~5	~28	[41]
NGS	0.2	high	Known + novel	~168	~1112	[42]

## Data Availability

Code will be made available on request.

## Supplementary Materials

The following supporting information can be downloaded at: https://www.mdpi.com/article/10.3390/bios15050308/s1, Table S1: LBDA detection of variant allele fractions (VAF) and mutation types in 22 KRAS-mutant samples. Figure S1: Comparison of CRC tissue (*n* = 120) and blood (*n* = 11) samples from the NGS results in Chinese population. (a) Top 10 gene mutation frequencies of the in CRC tissue samples. (b) Top 10 gene mutation frequencies of the in CRC blood samples. (c) Comparison of RAS mutation frequencies between CRC tissue and blood samples. Figure S2: Comparison of TCGA and Chinese cohorts. (a) Comparison of frequency of RAS mutation type between TCGA and Chinese cohorts. (b) The Venn diagram of mutations of TP53, APC, KRAS, and TTN in TCGA cohort. (c) The Venn diagram of mutations of TP53, APC, KRAS, and TTN in Chinese cohort. Figure S3: qPCR amplification curves of tumor and peritumoral samples from 3 KRAS non-mutated patients tested by LBDA. (a) Sample S56. (b) Sample S57. (c) Sample S58. Figure S4: Schematic of qPCR detection of tumor tissue samples from four clinical cases with (WB) and without (NB) blocker, showing the amplification curves. (a) Sample S8. (b) Sample S13. (c) Sample S19. (d) Sample S20. Figure S5: LBDA detection amplification curves of the S2 sample and Sanger sequencing chromatograms after qPCR amplification with blocker. Figure S6: LBDA detection amplification curves of the S14 sample and Sanger sequencing chromatograms after qPCR amplification with blocker. Figure S7: LBDA detection amplification curves of the S15 sample and Sanger sequencing chromatograms after blocker amplification. Figure S8: LBDA detection amplification curves of the S21 sample and Sanger sequencing chromatograms after qPCR amplification with blocker. Figure S9: LBDA detection amplification curves of the S23 sample and Sanger sequencing chromatograms after qPCR amplification with blocker. Figure S10: LBDA detection amplification curves of the S24 sample and Sanger sequencing chromatograms after qPCR amplification with blocker. Figure S11: LBDA detection amplification curves of the S25 sample and Sanger sequencing chromatograms after blocker amplification. Figure S12: qPCR amplification curves of tumor tissue samples from 11 KRAS mutation patients tested by LBDA. (a) Sample S6. (b) Sample S8. (c) Sample S33. (d) Sample S34. (e) Sample S37. (f) Sample S44. (g) Sample S46. (h) Sample S47. (i) Sample S53. (j) Sample S54. (k) Sample S55. Figure S13: Comparison of throughput, detection time, cost, and limit of detection between LBDA and NGS.
