# Supplementary material for: Analysis of Colorectal Cancer Gene Mutations and Application of Long Blocker Displacement Amplification Technology for High-Throughput Mutation Detection
Source: Biosensors (Basel). 2025 May 12;15(5):308. doi: 10.3390/bios15050308 (PMC12110167; doi:10.3390/bios15050308)
Supplement: Supplementary file 1 [file biosensors-15-00308-s001.zip › biosensors-3591629-supplementary.pdf]

biosensors
MDPI

Supplementary

Analysis of Colorectal Cancer Gene Mutations and Application of Long Blocker Displacement Amplification Technology for High-Throughput Mutation Detection

Ping Lu [1,2,†], Xinglei Su [3,4,5,†], Sirui Leong [3,†], Xuehao Xiu [3], Ping Song [3], Junjie Peng [1,2,*] and Yunpei Si [3,*]

[1] Department of Colorectal Surgery, Fudan University Shanghai Cancer Center, Fudan University, Shanghai 200032, China; hebenlu@163.com
[2] Department of Oncology, Shanghai Medical College, Fudan University, Shanghai 200032, China
[3] School of Biomedical Engineering, Zhangjiang Institute for Advanced Study and National Center for Translational Medicine, Shanghai Jiao Tong University, Shanghai 200240, China; suxinglei@shu.edu.cn (X.S.); jack23@sjtu.edu.cn (S.L.); xiuxuehao@sjtu.edu.cn (X.X.); songpingsjtu@sjtu.edu.cn (P.S.)
[4] Shanghai Key Laboratory for Nucleic Acid Chemistry and Nanomedicine Institute of Molecular Medicine Renji Hospital School of Medicine, Shanghai Jiao Tong University, Shanghai 200127, China
[5] School of Life Sciences, Shanghai University, Shanghai 200444, China
* Correspondence: pengjj@shca.org.cn (J.P.); yunpei@sjtu.edu.cn (Y.S.)
† These authors contributed equally to this work.

List of Tables

Table S1. LBDA detection of variant allele fractions (VAF) and mutation types in 22 KRAS-mutant samples.

List of Figures

Figure S1. Comparison of CRC tissue (*n*=120) and blood (*n*=11) samples from the NGS results in Chinese population.

Figure S2. Comparison of TCGA and Chinese cohorts.

Figure S3. qPCR amplification curves of tumor and peritumoral samples from 3 KRAS non-mutated patients tested by LBDA.

Figure S4. Schematic of qPCR detection of tumor tissue samples from four clinical cases with (WB) and without (NB) blocker, showing the amplification curves.

Figure S5. LBDA detection amplification curves of the S2 sample and Sanger sequencing chromatograms after blocker amplification.

Figure S6. LBDA detection amplification curves of the S14 sample and Sanger sequencing chromatograms after blocker amplification.

Figure S7. LBDA detection amplification curves of the S15 sample and Sanger sequencing chromatograms after blocker amplification.

Figure S8. LBDA detection amplification curves of the S21 sample and Sanger sequencing chromatograms after blocker amplification.

Figure S9. LBDA detection amplification curves of the S22 sample and Sanger sequencing chromatograms after blocker amplification.

Figure S10. LBDA detection amplification curves of the S23 sample and Sanger sequencing chromatograms after blocker amplification.

Figure S11. LBDA detection amplification curves of the S24 sample and Sanger sequencing chromatograms after blocker amplification.

Figure S12. qPCR amplification curves of tumor tissue samples from 11 KRAS mutation patients tested by LBDA.

Figure S13. Comparison of throughput, detection time, cost, and limit of detection between LBDA and NGS.

Table S1. LBDA detection of variant allele fractions (VAF) and mutation types in 22 KRAS-mutant samples.

Sample	VAF (%)	DNA Mutation	AA Mutation
S2	0.55%	c.35G>T	P.G12V
S3	0.49%	c.35G>A	P.G12D
S6	0.78%	c.38G>A	P.G13D
S8	1.76%	c.35G>T	P.G12V
S13	11.00%	c.35G>A	P.G12D
S14	0.63%	c.38G>A	P.G13D
S15	0.46%	c.35G>C	P.G12A
S19	1.21%	c.38G>A	P.G13D
S20	11.47%	c.34G>T	P.G12C
S21	8.64%	c.34G>A	P.G12S
S23	5.28%	c.38G>A	P.G13D
S24	13.17%	c.38G>A	P.G13D
S25	16.72%	c.35G>A	P.G12D
S33	1.66%	c.35G>C	P.G12A
S34	9.90%	c.38G>C	P.G13A
S37	78.58%	c.34G>T	P.G12C
S44	0.43%	c.38G>A	P.G13D
S46	94.30%	c.35G>A	P.G12D
S47	1.51%	c.35G>T	P.G12V
S53	6.31%	c.37G>T	P.G13C
S54	2.58%	c.34G>A	P.G12S
S55	1.27%	c.35G>A	P.G12D

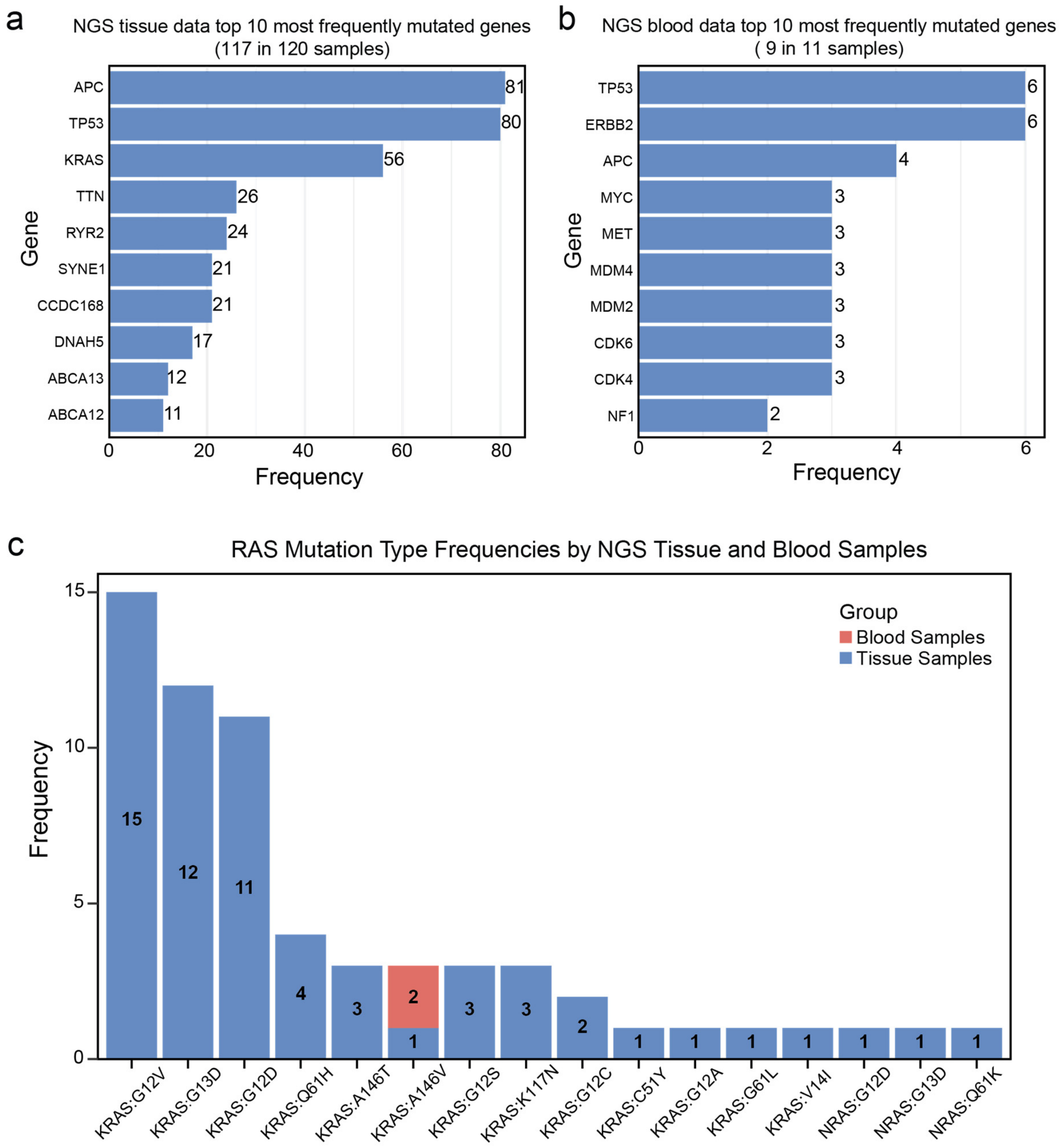

Figure S1. Comparison of CRC tissue (*n*=120) and blood (*n*=11) samples from the NGS results in Chinese population. (a) Top 10 gene mutation frequencies of the in CRC tissue samples. (b) Top 10 gene mutation frequencies of the in CRC blood samples. (c) Comparison of RAS mutation frequencies between CRC tissue and blood samples.

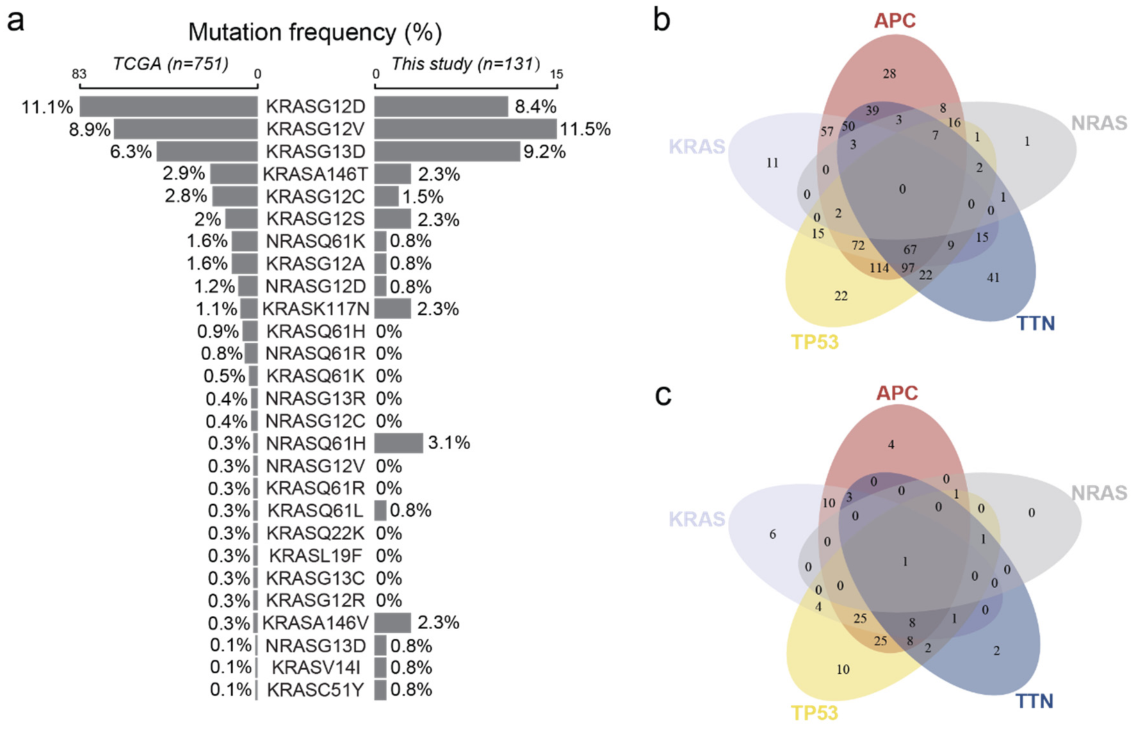

Figure S2. Comparison of TCGA and Chinese cohorts. (a) Comparison of frequency of RAS mutation type between TCGA and Chinese cohorts. (b) The Venn diagram of mutations of TP53, APC, KRAS, and TTN in TCGA cohort. (c) The Venn diagram of mutations of TP53, APC, KRAS, and TTN in Chinese cohort.

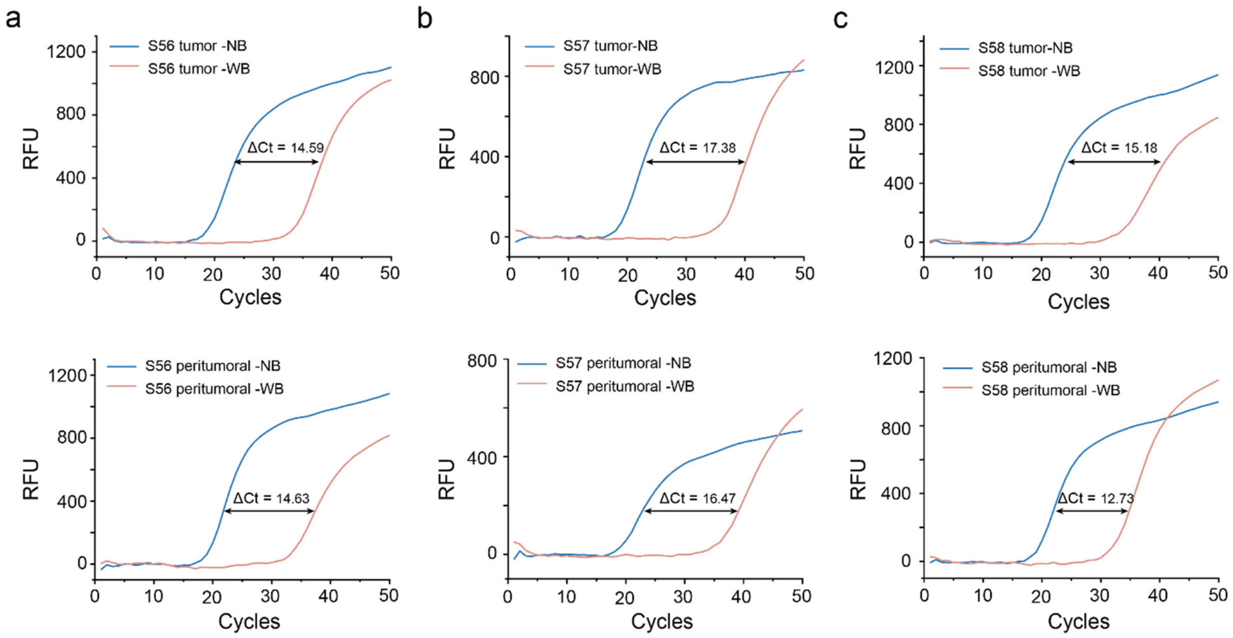

Figure S3. qPCR amplification curves of tumor and peritumoral samples from 3 KRAS non-mutated patients tested by LBDA. (a) Sample S56. (b) Sample S57. (c) Sample S58.

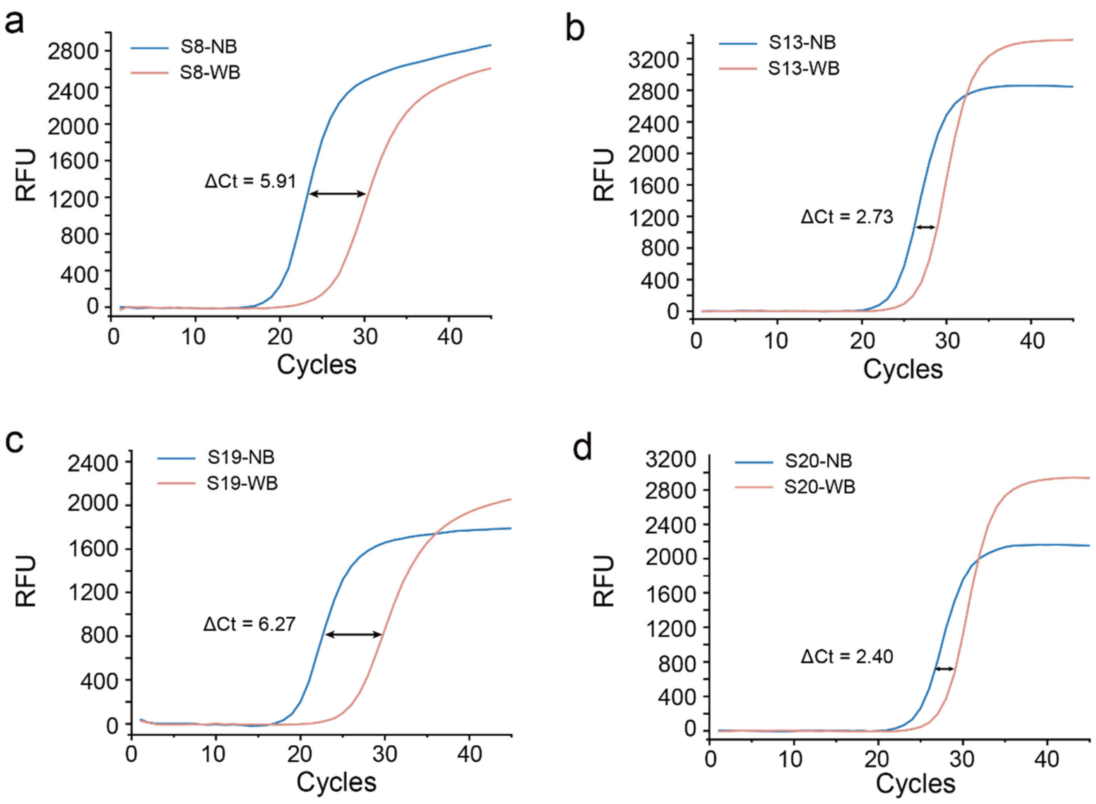

Figure S4. Schematic of qPCR detection of tumor tissue samples from four clinical cases with (WB) and without (NB) blocker, showing the amplification curves. (a) Sample S8. (b) Sample S13. (c) Sample S19. (d) Sample S20.

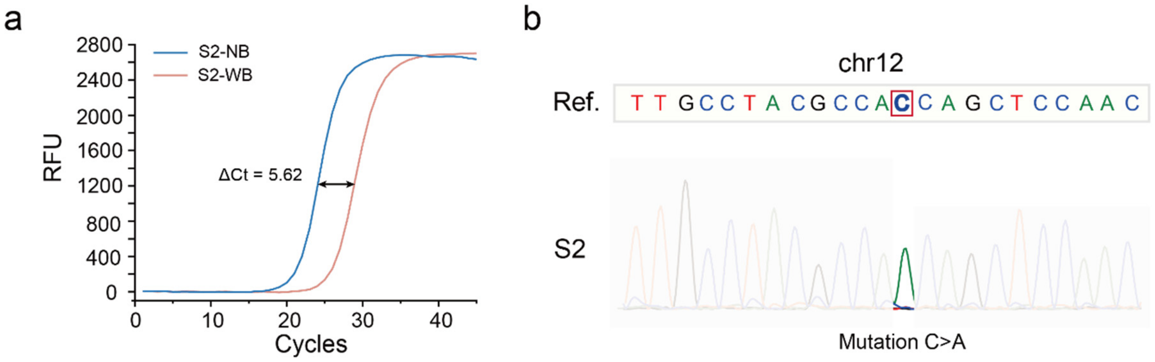

Figure S5. LBDA detection amplification curves of the S2 sample and Sanger sequencing chromatograms after qPCR amplification with blocker.

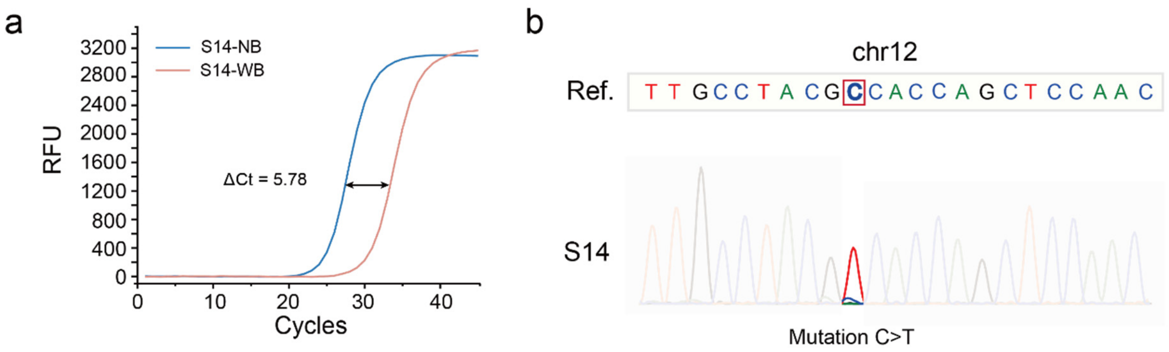

Figure S6. LBDA detection amplification curves of the S14 sample and Sanger sequencing chromatograms after qPCR amplification with blocker.

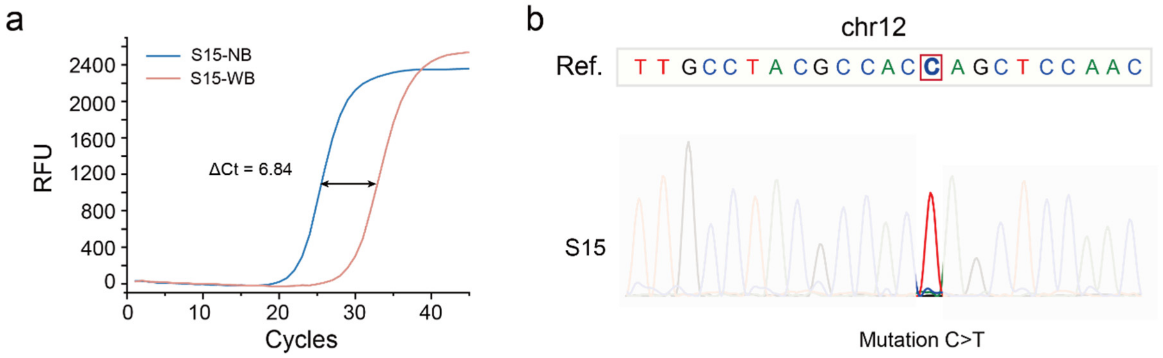

Figure S7. LBDA detection amplification curves of the S15 sample and Sanger sequencing chromatograms after blocker amplification.

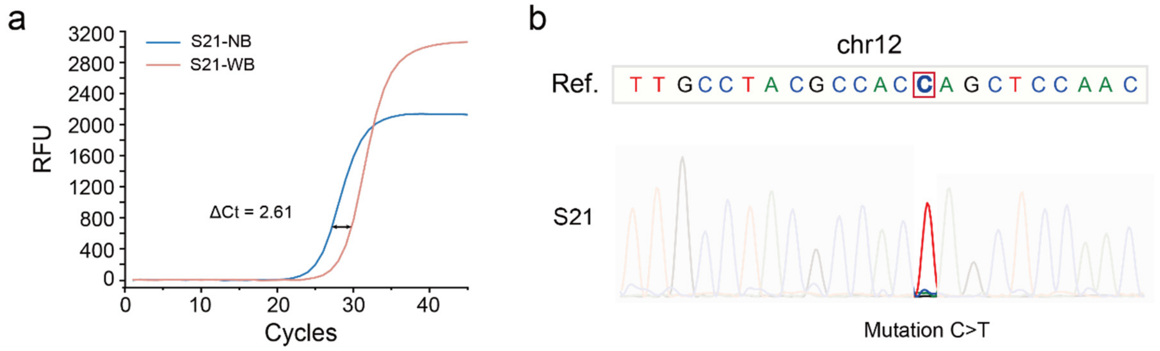

Figure S8. LBDA detection amplification curves of the S21 sample and Sanger sequencing chromatograms after qPCR amplification with blocker.

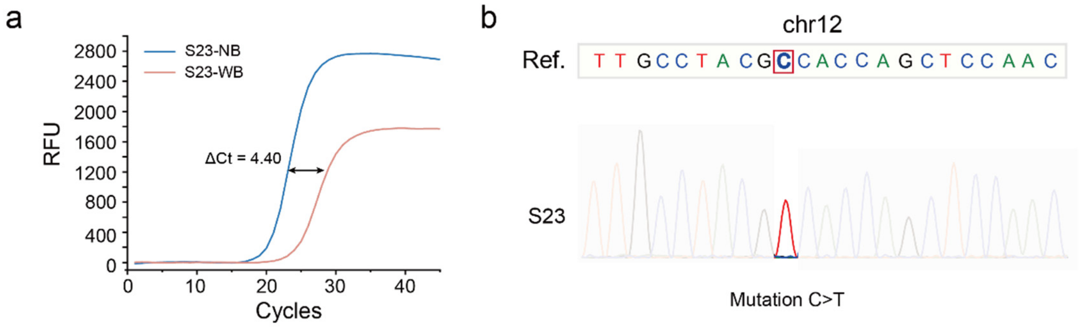

Figure S9. LBDA detection amplification curves of the S23 sample and Sanger sequencing chromatograms after qPCR amplification with blocker.

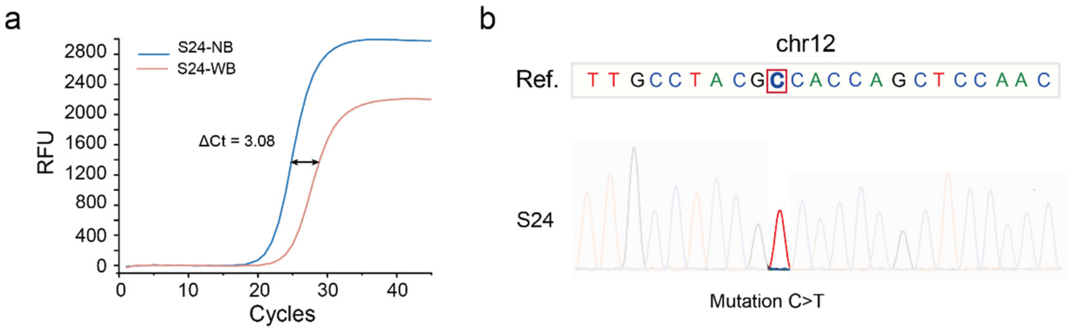

Figure S10. LBDA detection amplification curves of the S24 sample and Sanger sequencing chromatograms after qPCR amplification with blocker.

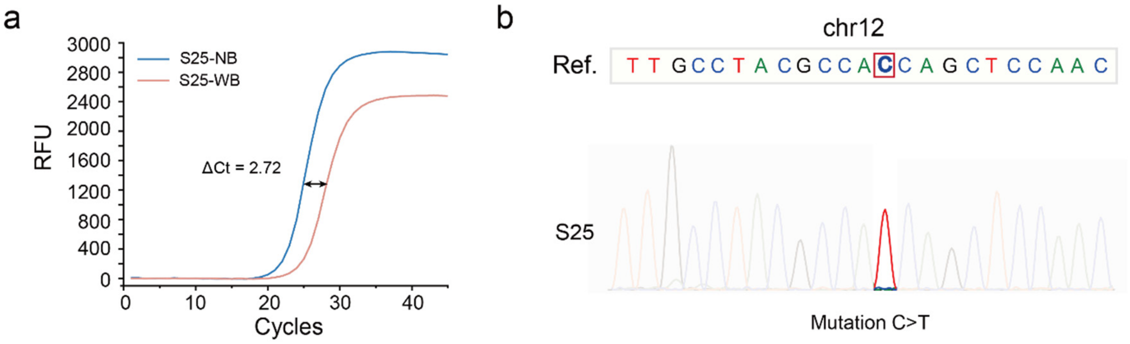

Figure S11. LBDA detection amplification curves of the S25 sample and Sanger sequencing chromatograms after blocker amplification.

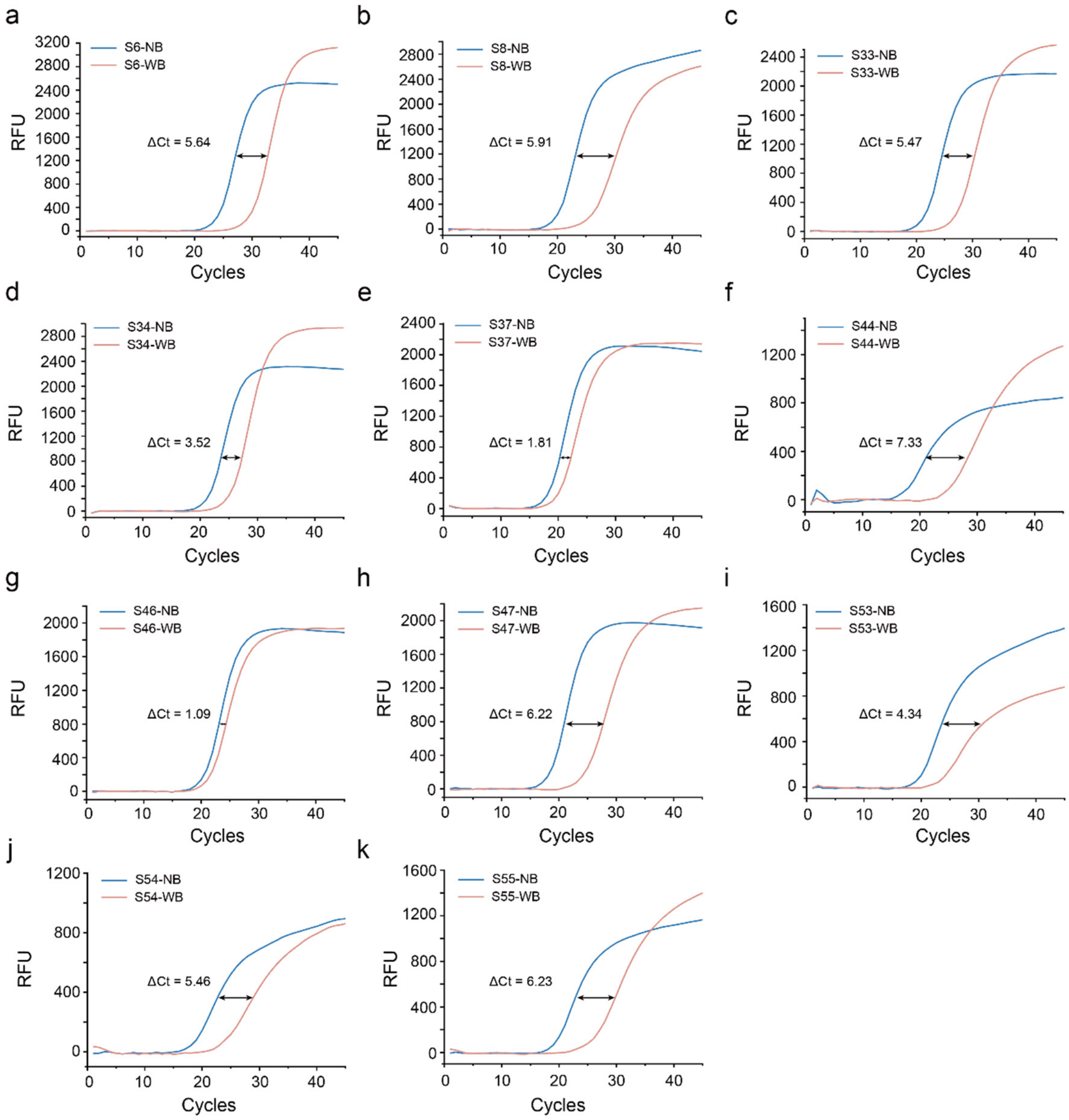

Figure S12. qPCR amplification curves of tumor tissue samples from 11 KRAS mutation patients tested by LBDA. (a) Sample S6. (b) Sample S8. (c) Sample S33. (d) Sample S34. (e) Sample S37. (f) Sample S44. (g) Sample S46. (h) Sample S47. (i) Sample S53. (j) Sample S54. (k) Sample S55.

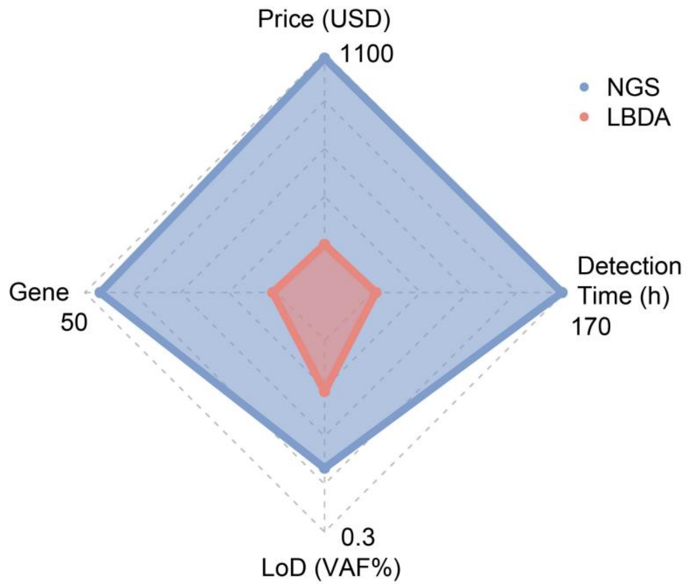

Figure S13. Comparison of throughput, detection time, cost, and limit of detection between LBDA and NGS.
